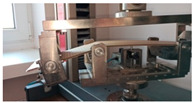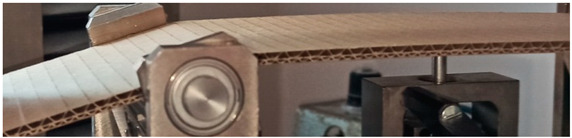# Numerical and Experimental Study of Five-Layer Non-Symmetrical Paperboard Panel Stiffness

**DOI:** 10.3390/ma14237453

**Published:** 2021-12-04

**Authors:** Leszek Czechowski, Gabriela Kmita-Fudalej, Włodzimierz Szewczyk, Jacek Gralewski, Maria Bienkowska

**Affiliations:** 1 Department of Strength of Materials, Lodz University of Technology, 90-537 Lodz, Poland; 2 Centre of Papermaking and Printing, Lodz University of Technology, 93-005 Lodz, Poland; gabriela.kmita-fudalej@dokt.p.lodz.pl (G.K.-F.); wlodzimierz.szewczyk@p.lodz.pl (W.S.); maria.bienkowska@p.lodz.pl (M.B.); 3 Institute of Social Sciences and Management of Technologies, Lodz University of Technology, 90-924 Lodz, Poland; jacek.gralewski@p.lodz.pl

**Keywords:** stiffness of paperboard, bending tests, mechanical properties

## Abstract

This paper concerns the analysis of five-layer corrugated paperboard subjected to a four-point bending test. The segment of paperboard was tested to determine the bending stiffness. The investigations were conducted experimentally and numerically. The non-damaging tests of bending were carried out in an elastic range of samples. The detailed layers of paperboard were modelled as an orthotropic material. The simulation of flexure was based on a finite element method using Ansys^®^ software. Several material properties and thicknesses of papers in the samples were taken into account to analyse the influence on general stiffness. Two different discrete models based on two geometries of paperboard were considered in this study to validate the experimental stiffness. The present analysis shows the possibility of numerical modelling to achieve a good correlation with experimental results. Moreover, the results of numerical estimations indicate that modelling of the perfect structure gives a lower bending stiffness and some corrections of geometry should be implemented. The discrepancy in stiffness between both methods ranged from 3.04 to 32.88% depending on the analysed variant.

## 1. Introduction

The essential role of packages is a kind of protection of products from their failure before they are delivered to customers, therefore packages should be simultaneously stiff and light. Based on this assumption, many packages are made of paper or of paperboard to satisfy these restrictions and moreover to be recyclable and biodegradable. One of the most important issue for economical packaging is designing an optimal structure characterised by high mechanical properties with respect to weight. Three- and five-layer corrugated paperboard is a popular material for the production of transport packaging used in many branches of industry and commerce. Five-layer corrugated paperboard is distinguished by much better strength properties in comparison to three-layer corrugated paperboard. Transport boxes are stacked during transport and stored, therefore their high resistance to static pressure is necessary to ensure that the packed goods are properly secured. The static pressure resistance of boxes can be predicted from the dimensions of the box and its strength properties, which include bending stiffness. Predicting the box compression test (BCT) allows one to economically select the type of corrugated paperboard that meets the requirements of the boxes. While measuring the BCT, one can observe a tendency for the box walls to give out in the outward direction. This effect can be controlled by an appropriate arrangement of five-layer corrugated paperboard with waves of different heights and by a strength increase of the box. In the literature, there have been many recent works related to the strength of papers or paperboard. The authors of [1,2,3] investigated numerically and experimentally the strength and creeping of paperboard boxes in different conditions due to compression. Mathematical models for predicting the stress peaks of corrugated paperboards were considered in [4]. Bai et al. in [5] examined the behaviour of single corrugated paperboard under axial crushing. Zaheer et al. in [6] assessed the strength of compressed paperboard box by employing a finite element method. The authors of [7] tested the creasing and the folding of three paperboards. Wallmeier et al. in [8] analysed the damage of deep-drawn paperboard cups. The studies on the strength of paper tubes under lateral loads were conducted in [9,10]. Other works directly linked to numerical experiments of corrugated cardboard strength were shown in [11,12,13,14,15]. Results relating the study of the honeycomb cardboard’s strength were included in [16,17,18,19]. Based on the first-order shear deformation theory Hernández-Pérez et al. in [20] analysed the twist stiffness of single- and double-wall corrugated board. Hämäläine et al. in [21] examined the transverse shear properties of paper due to a short span compression test. Bolzon and Talassi in [22] assessed the behaviour of anisotropic paperboard composites by driving them to their damage point by means of burst strength testers. Mentrasti et al. studied the behaviour of creased paperboard experimentally in [23] and analytically in [24]. The authors of [25] investigated the flexural damage of honeycomb paperboard based on FEM and experimental tests. On the other hand, the research on composite material structures within a buckling and a post-buckling state were carried out numerically in [26,27,28,29] and/or experimentally in [30,31,32,33,34,35,36,37]. Progressive failure analysis in numerical computations was considered in papers [38,39,40], among others. In [41], Minh presented an analytic homogenization model for the 5-layer corrugated paperboard under transverse loading. The homogenization was carried out by calculating analytically the global rigidities of the 5-layer corrugated paperboard and then a 3-dimensional (3D) structure was replaced by an equivalent homogenized 2-dimensional (2D) plate. In simulations, the transverse loading used Abaqus-3D and H-2D models. The subject of simulation was a 5-layer paperboard with wave heights of 5.2 mm and 2.9 mm and period (or step) of 9 mm and 6 mm, respectively, only the flat layer adjacent to the higher wave was compressed during bending. During the simulation, no local deformations (buckling) between peaks of the waves in bending in the machine direction were present, and the stiffness simulation was not performed using the opposite bending moment. The authors of [42] carried out extended laboratory tests on the crushing of 5-layer corrugated paperboard to study the influence of fully controlled crushing (with a precision of ±10 μm). The range of corrugated board crushing was from 10 to 70% of its initial thickness with increments of 10%. Most of the typical mechanical tests were performed, e.g., edge crush test, four-point bending test, shear stiffness test, torsional stiffness test, etc., on reference and crushed specimens. Four types of 5-layer corrugated paperboard were analysed, two with waves BC and two with waves EB. All empirical observations and performed measurements were the basis for building an analytical model of crushed corrugated board. A 3D structure was replaced by a homogenized 2D plate. The proven and verified model was then used to study the crushing effect of the selected corrugated board on typical mechanical tests and the efficiency of simple packages with various dimensions. In [43], Han et al. studied the strength of the adhesively bonded corrugated sandwich beams due to a three-point bending test.

Based on the aforementioned literature, one can find a few papers directly linked to strength/stiffness especially of one-walled corrugated paperboards. The present investigation deals with experimental and numerical analysis of double-wall corrugated paperboard subjected to a four-point flexural test. Experimental tests were carried out for different thicknesses of skins and of corrugated paper to determine their stiffnesses. Moreover, two types of numerical models (without homogenization) were taken into account to validate the experimental results. The tests were conducted until local deformations (buckling) appeared. The simulations were performed in Ansys^®^ version 18.2 [44] for large displacements relying on the Green–Lagrange equations. An assessment of different thicknesses of walls of corrugated paperboard and a validation of numerical models can be useful to conduct further simulations on more complicated structures.

## 2. Problem Formulation

### 2.1. Object of Analysis

**Figure 1:**
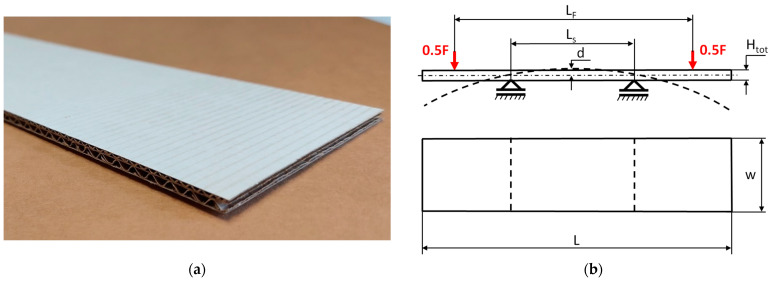
View of paperboard panel part (**a**) and scheme of support with dimensions of sample (**b**).

The object of analysis was double-wall corrugated paperboard. Such paperboards are produced in the form of panels presented in Figure 1a. According to Figure 1b, the dimensions of the panel are as follows: total length *L* = 500 mm, width *w* = 100 mm and *H*_tot_ ranges from 3.911 to 4.344 mm in reference to the considered model. The panel of corrugated paperboard was subjected to 4-point bending tests where the distance between spans denoted as *L*_s_ amounted to 200 mm and that between symmetrically applied forces *L*_F_ was equal to 400 mm. During the tests, the deflection d (mid-deflection) was measured.

**Figure 2:**
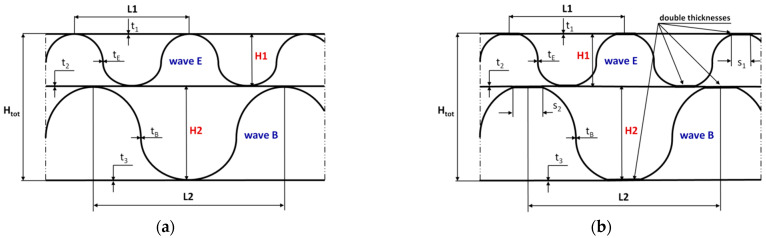
The geometry and dimensions of analysed paperboard for GEOM_1 (**a**) and GEOM_2 (**b**).

For the numerical analysis, two different models were considered (GEOM_1 and GEOM_2). The first one shown in Figure 2a was based on the purely theoretical assumption that the connection between the waves and flat surfaces occurred only linearly (this corresponds to a perfect structure). The second one concerned the building of paperboard closer to a real one, as presented in Figure 1a. In this case, the connection between the waves and remaining parts was considered on some surfaces with constant widths denoted *S*_1_ and *S*_2_ (Figure 2b). Figure 2a,b show the magnitudes as thicknesses *t*_1_, *t*_2_, *t*_3_, *t*_E_ and *t*_B_ or heights of waves *H1* and *H2* describing the geometry of the double-wall corrugated paperboard. The height of minor wave *H1* and major wave *H2* was equal to 1.20 mm and 2.58 mm, respectively. The distances *L1* and *L2* for wave E and wave B amounted to 3.4 mm and 6.1 mm, respectively. The corrugation coefficients were 1.262 (wave E) and 1.362 (wave B). In the case of glued surfaces, *S*_1_ and *S*_2_ were equal to 0.64 mm and 0.94 mm, respectively.

### 2.2. Material Properties

**Figure 3:**
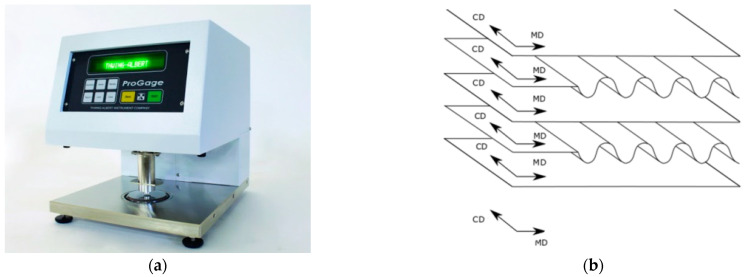
The device for thickness measurements (**a**) and orientation of MD and CD (**b**).

Before manufacturing the paperboard samples, the thicknesses of the detailed layer were measured on the thickness device ProGage (New Jersey, USA) (Figure 3a). The mean thicknesses given in Table 1 were obtained from 20 measures for each paper. The static pressure on a surface of 200 mm^2^ at the paper measurements was equal to 100 kPa. The measures of thickness of the paper attained an accuracy of 0.001 mm. The material of the paper was assumed to be orthotropic and linear. The Young’s moduli for the machine direction (MD) and cross direction (CD) of each model were determined by using one-directional tensile tests.

The orientation of MD and CD is depicted in Figure 3b. The values of moduli *E*_MD_, *E*_CD_ inserted into Table 1 are mean values from 10 separate measures. The Poisson’s ratios were taken from the literature, but the shear modulus *G*_MD-CD_ was calculated based on the formula (0.33·*E*_MD_·*E*_CD_)^0.5^.

### 2.3. Test Stand

**Figure 4:**
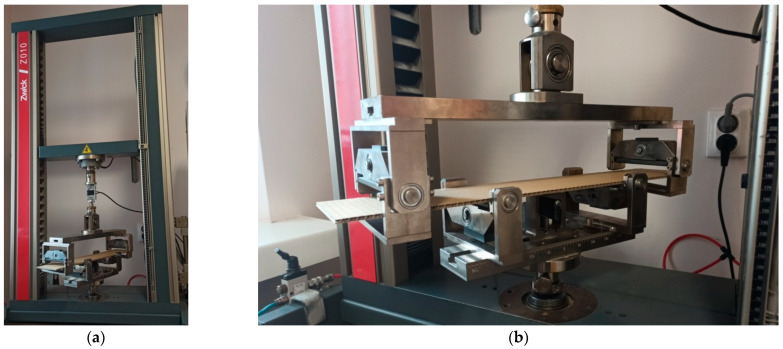
The test stand for 4-point bending test (**a**) equipped with special grip (**b**).

Before the tests of bending, the paperboard samples were dried at a temperature of 40 °C and then they were conditioned according to standard PN-EN 20187:2000 (temperature 23 ± 1 °C and relative air humidity 50 ± 2%) [44]. These bending tests were conducted on a Zwick Tensile Machine model Z010 (Ulm, Germany) equipped with a specialized tool (Figure 4a). The load range of the machine was from 0.1 N to 10 kN. During bending tests, the jaw of the machine was moving with a velocity of 10 mm/min. The method of placing the sample in the measuring grip is shown in Figure 4b. According to the standard used, the bending tests were carried out in two stages of loading: the first one was based on preload (5–10% range of maximum load) and the second one included the moment until large deformations occurred (local buckling between peaks of the waves).

### 2.4. Bending Stiffness

Bending stiffness (*BS*) is one of the most important coefficients applied in papermaking. This magnitude is determined by the equation given below (Equation (1)). The *BS* based on the dimensions from Figure 1b is expressed in Nm. *BS* ranges from 10 to 40% of the full loading before large deformations occur (linear scope of bending curve).

(1) $$BS=\frac{F\cdot L_{S}^{2}\cdot (L_{F}-L_{S})}{32\cdot w\cdot d}$$

### 2.5. FE Models

**Figure 5:**
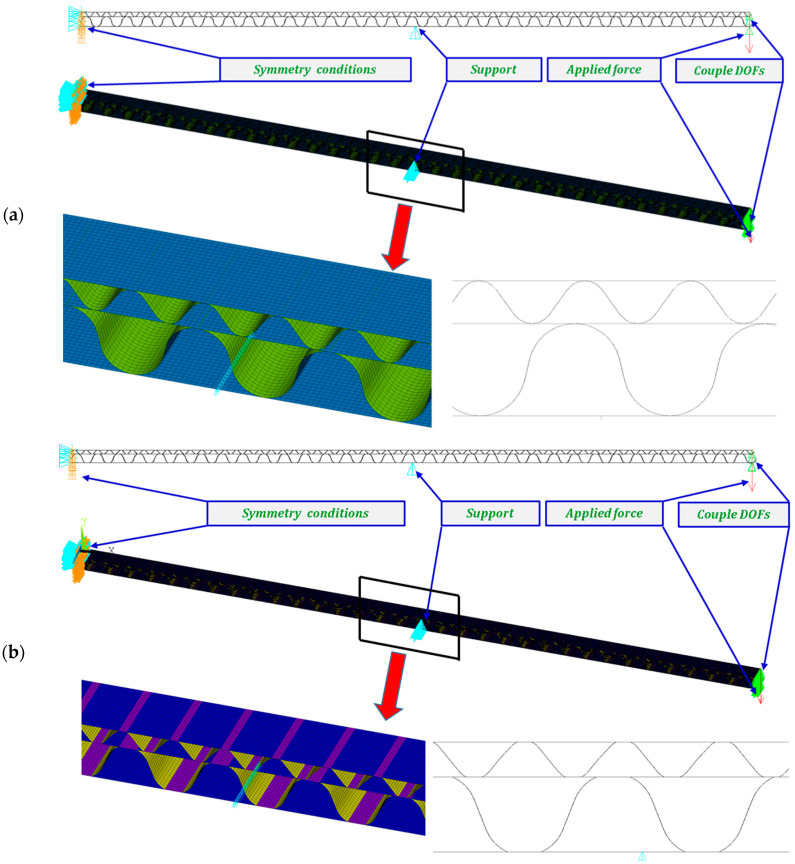
FE discrete models with boundary conditions: GEOM_1 (**a**) and GEOM_2 (**b**).

A numerical simulation was performed for a half of the panel (200 mm) at a width of 5 mm in Ansys^®^ software version 18.2 [45]. The diminished width of the panel resulted from a necessity to limit the number of finite elements but for a 4-point bending simulation it did not play a great role. Finally, to refer to empirical result, the total bending force was multiplied by 40. In the analysis, symmetry conditions were applied for both models (Figure 5a—GEOM_1 and Figure 5b—GEOM_2). The discrete models were created by using the 4-node shell 181 element. Based on the software description [45], this element is useful to analysing thin or moderately thick shell structures. The directions of the paper orthotropy correspond to the orientation given in Figure 3b. In the case of GEOM_2, the touching walls between flat and corrugated paper were modelled as double thicknesses (see Figure 5b). The size of a finite element was assumed to be 0.2–0.25 mm, therefore each numerical model was built of more than 100,000 finite elements. The realisation of loading was executed by applying force to one node. This node was connected to the outer nodes of the panel to cause a common vertical displacement (preventing the local deformation of the panel thanks to the application of couple DOFs option). The simulations were conducted based on large displacements of the Green–Lagrange equations. The nonlinear estimation runs carried out in substeps relied on the Newton–Raphson algorithm. The number of substeps was assumed to be from 100 to 800, however, the number of iterations in each substep was set between 10 to 5000. Such a setting enabled us to achieve the convergence of the calculations.

## 3. Results

### 3.1. Stiffness Analysis

**Figure 6:**
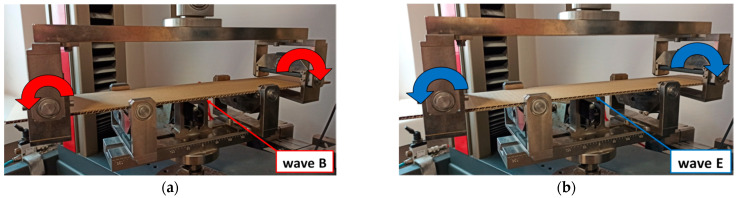
Mid-deflection vs. force for Model_1 with compressed B-wave (**a**) and E-wave (**b**).

**Figure 7:**
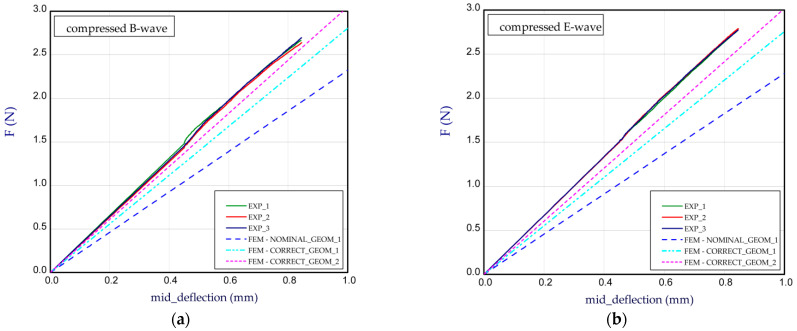
Mid-deflection vs. force for Model_1 with compressed B-wave (**a**) and E-wave (**b**).

**Figure 8:**
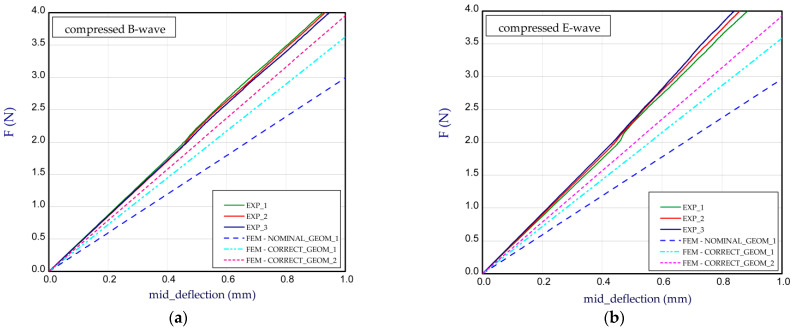
Mid-deflection vs. force for Model_2 with compressed B-wave (**a**) and E-wave (**b**).

**Figure 9:**
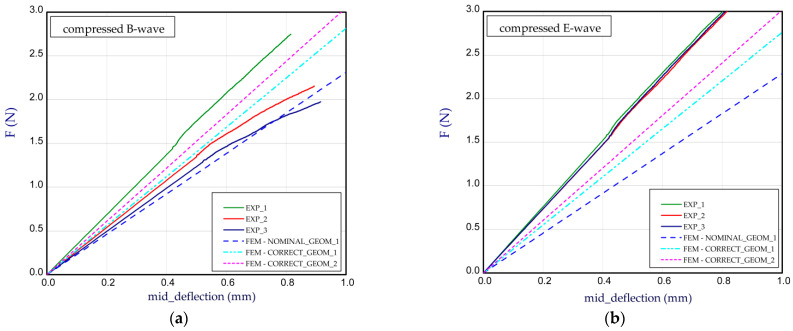
Mid-deflection vs. force for Model_3 with compressed B-wave (**a**) and E-wave (**b**).

**Figure 10:**
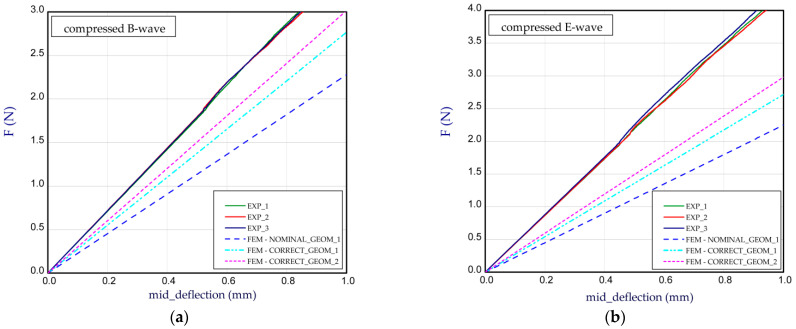
Mid-deflection vs. force for Model_4 with compressed B-wave (**a**) and E-wave (**b**).

**Figure 11:**
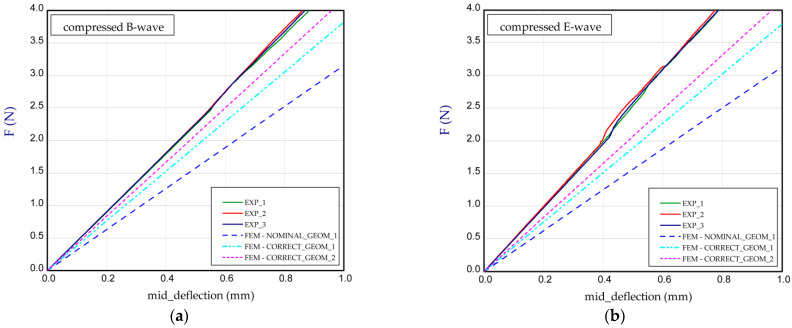
Mid-deflection vs. force for Model_5 with compressed B-wave (**a**) or E-wave (**b**).

**Figure 12:**
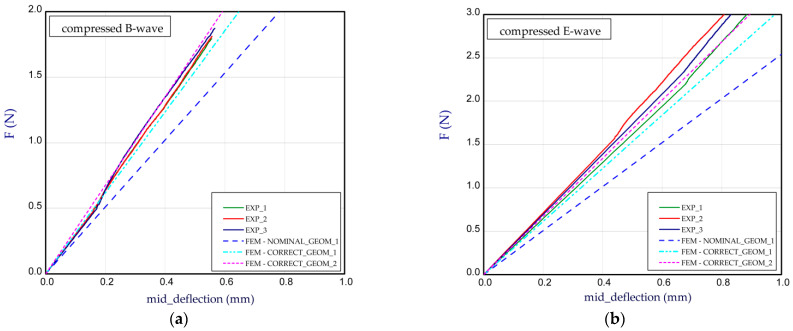
Mid-deflection vs. force for Model_6 with compressed B-wave (**a**) or E-wave (**b**).

This subsection presents the results of the bending of all the models. Two arrangements of samples were considered: a compressed B-wave on the bottom (Figure 6a) or a compressed E-wave on the bottom (Figure 6b). It means that by reversing the sample direction, the applied moment is different. We considered two cases from our observation during the experiment that the stiffness of panels with regard to non-symmetrical waves could slightly differ from each other; therefore, a further analysis included both ways of moment application. In Figure 7, Figure 8, Figure 9, Figure 10, Figure 11 and Figure 12, the charts are expressed as full bending force F vs. mid-deflection (d). The experimental tests were done for three samples of each model without causing damage.

Figure 7a presents the bending curves for Model_1 by a compressed B-wave (the B-wave in the paperboard is determined through greater height). In this case, the thickness of the papers used for the waves is equal (*t*_E_ = *t*_B_) and the thicknesses of the papers used for the flat layers of corrugated paperboard *t*_1_, *t*_2_, *t*_3_ are equal to 0.142 mm, 0.126 mm and 0.146 mm, respectively. Numerical simulations were conducted for a nominal height of the panel (NOMINAL_GEOM_1, then *H*_tot_ = 3.911 mm). It means that the thicknesses of the papers used for the flat layers according to Table 1 and the wave heights E-wave and B-wave were taken into account. We see that the stiffness for NOMINAL_GEOM_1 is significantly smaller than that obtained in the experiment. It can result from the fact that the nominal height (denoted as NOMINAL) of the panel was based on the thin-walled structures built of mid-surfaces [45]. It means that the effective thickness of the panel is usually slightly lower than the thickness of the real model. Therefore, by increasing the height of the panel (CORRECT_GEOM_1 with *H*_tot_ = 4.302 mm) in order to achieve an almost real thickness of panel, the stiffness rose by 10–20%, but this result is still lower in reference to the experimental one. Based on the results of CORRECT_GEOM_2 (*H*_tot_ = 4.302 mm), a good consistency with the experiment was obtained. It also seems that this approach is more appropriate. Looking at Figure 7b, while the E-wave of the panel was compressed, a small growth in stiffness was observed but this was apparent only for the experimental results. The numerical results of the bending for the compressed E-wave in contrast to the empirical ones did not indicate a substantial change. The bending curves in the simulation seem to be the same regardless of the direction of the moment application. In the case of the compressed E-wave (Figure 7b), a higher discrepancy is noticed between experiment and CORRECT_GEOM_2.

It can be also mentioned that the experimental curves for Model_1 are very comparable. The *BS* estimated for all variants are presented in Table 2. The next diagram (Figure 8) concerns the panel with other thicknesses of paper (see Table 1, Model_2). The general thicknesses of paper are close to 0.2 mm and a slight difference in thickness between papers used for the E-wave and B-wave exists. For Model_2, the thickness of the paper for the E-wave is greater than that for the B-wave. The trends of the curves are similar to those of the curves for Model_1, but for the compressed E-wave, the experimental stiffness is decidedly higher, even for numerical CORRECT_GEOM_2 with *H*_tot_ = 4.344 mm (for NOMINAL_GEOM_1, *H*_tot_ = 3.949 mm). Moreover, the obtained stiffness of CORRECT_GEOM_1 (Figure 8b) is slightly lower than the stiffness of CORRECT_GEOM_2 with *H*_tot_ = 4.344 mm (Figure 8a). In general, this effect was reverse in the experiment. The greater discrepancies are observed for Model_3 (Figure 9). The experimental curves for the B-wave significantly differ from each other but the numerical curves are between them (for NOMINAL_GEOM_1, *H*_tot_ = 3.911 mm). A weak repeatability can result from the samples defect or an initial deformation that led to achieve nonlinear characteristics. In this considered case, the thickness of the paper for the E-wave is greater than that for the B-wave.

More regular trends of experimental curves are seen in terms of compressed E-wave (Figure 9b), but numerical results are still lower in comparison to the experimental ones. The curves of CORRECT_GEOM_1 (*H*_tot_ = 4.302 mm) and CORRECT_GEOM_2 (*H*_tot_ = 4.302 mm) go similarly but the latter one is closer to the empiric one. Hence, overall, the numerical approach using CORRECT_GEOM_2 gives better results.

By taking into consideration Model_4 (*t*_E_ = *t*_B_, for NOMINAL_GEOM_1, *H*_tot_ = 3.911 mm and CORRECT_GEOM_1, *H*_tot_ = 4.302 mm), a similar effect was observed; nevertheless, a greater difference for the case of the compressed E-wave appeared (Figure 10). Numerically obtained relations for Model_4 in reference to the experiment are similar to those of Model_1 (both these models have equal thicknesses of paper for the E-wave and B-wave). Considering the charts from Figure 11 for Model_5, the discrepancy in the paperboard stiffness for CORRECT_GEOM_2 (*H*_tot_ = 4.344 mm) amounted to about 9% and 20% for compressed B-wave and E-wave, respectively. In this case, we also have *t*_E_ = *t*_B_ (thicknesses of the waves) and that the thicknesses of the flat paper are almost the same as the remaining thicknesses, because the ratios are *t*_1_/*t*_E_ = 0.930 and *t*_3_/*t*_B_ = 0.935. In the previous case (Model_4), the ratios were: *t*_1_/*t*_E_ = 0.802 and *t*_3_/*t*_B_ = 0.824. It means that at greater thicknesses of the walls, the numerical model better corresponds to experiment results.

The bending curves for Model_6 are illustrated in Figure 12 (NOMINAL_GEOM_1, *H*_tot_ = 3.929 mm and CORRECT_GEOM_1, *H*_tot_ = 4.322 mm). Based on the results from Table 2, the stiffness of paperboard is very comparable with that of the experiment. Moreover, the numerical solution gave a slightly higher score in the case of the B-wave compression and a lower score by 7% for the E-wave compression. In this case, the thickness ratios are: *t*_E_ = *t*_B_, *t*_1_/*t*_E_ = 0.802 and *t*_3_/*t*_B_ = 1.050. Based on the *BS* of all considered paperboard variants, the highest stiffnesses were noted for Model_2 and Model _5. It results from the fact that for these case configurations the thicknesses in the paperboard are the greatest.

### 3.2. Deformations Maps

The results of deformations maps in full range of bending for Model_1 are plotted in Table 3 and Table 4. The maps show sequentially the behaviour of a paperboard segment under different forces for two analysed geometries in the case of a compressed B-wave (Table 3) and a compressed E-wave (Table 4). The scale given in the legend is given in millimetre. In the case of larger forces (e.g., line 5, *F* = 8 N), there is visible local deformation between the peaks of the B-wave. In general, this phenomenon is not an issue for the present paper but it seems necessary to show some effects which can occur when bending such structures. It means that apart from the thicknesses, the length between the wave peaks in the paperboard determines the maximum bending load. This effect at these forces was not noticed in the case of the compressed E-wave (Table 4). Of course, in that case, some point of buckling load is possible but a load causing local buckling would surely be higher.

### 3.3. Full Experimental Curves

**Figure 13:**
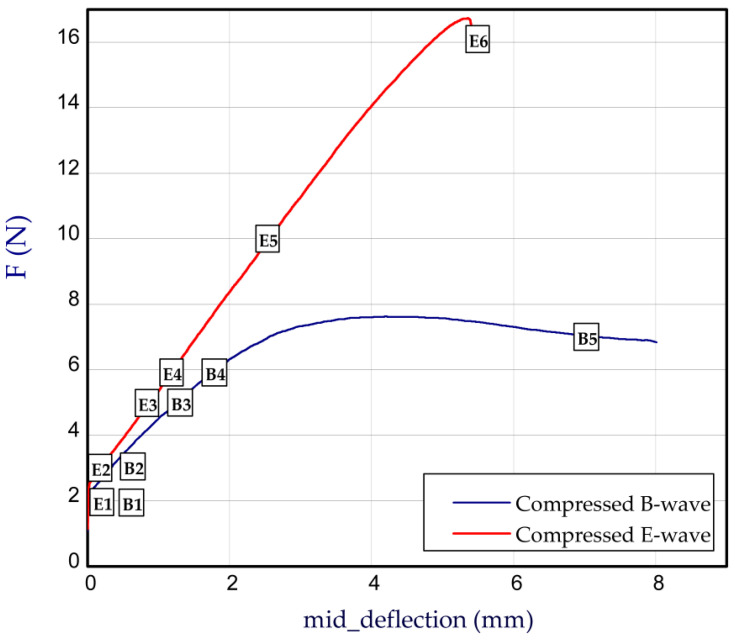
Full work curves for compressed B-wave and E-wave.

To look over the whole analysis of a single bending test, the exemplary characteristics with a full range (till the moment of panel flexure) are illustrated in Figure 13. It can be easily observed that the differences between the compressed B-wave and E-wave are distinct. The attained curves are discussed based on acquired pictures in in the following. The behaviour of the paperboard sample under different force values (based on Figure 13) in the case of compressed B-wave (Table 5) and compressed E-wave (Table 6) is shown in turn. Looking at Table 5, a small change at force 2 N (point B1) is visible. Subsequently, with the compressed B-wave at force 3 N (point B2), a local deformation of the compressed layer of flat corrugated cardboard between the peaks of the B-wave is seen. As the force value increases to 6 N (point B4), the local deformation increases and at a force value of about 7 N (point B5), the cardboard sample breaks between the inner supports of the special grip. For the E-wave compression in a range of force between 2 N and 10 N (points E1–E5), this effect is not noticed in the case of flat cardboard adjacent to the E-wave. The breaking force appears approximately at 16.5 N (point E6) and the failure point is observed at the support of the special grip.

## 4. Conclusions

This work concerns the analysis of four-point bending of five-layer non-symmetrical paperboard. The experimental and numerical experiment was done for six models characterized by different thicknesses of paper. The total height of paper could slightly differ with respect to other thicknesses of paper. Based on the results, it can be stated:

- The assumption of nominal thicknesses of the paper provides the lower stiffnesses in reference to those obtained in the experiment. Moreover, implementations of perfect structures with corrected thicknesses of paperboard also do not correlate well.
- The numerical results for perfect structures do not show the differences in *BS* between compressed B-wave and compressed E-wave.
- The measure results indicate a slight difference between the values of *BS* obtained at different signs of moments when bending paperboard in the machine direction. When an upper layer glued to the E-wave is compressed, the *BS* is higher in comparison to the *BS* of a compressed layer at the B-wave. This can be explained by a local deflection between joints (connection between waves and flat layers) that is greater if wave pitches are greater as well.
- The course of the curve seen in Figure 13 is characterized by a mild change between the initial part of chart (where compression dominates) and the field where buckling of the compressed plate occurs.
- We showed that numerically analysing of the behaviour of multilayer paperboard panels by including the aforementioned imperfections can reveal distinct differences in the *BS* for different signs of moment (in the case of compressed B-wave and E-wave). The lack of visible border in the results of the numerical simulation might just be caused by preliminary deflections.
- Depending on the analysed variant and arrangement of the panel, the mean values from the experiment were slightly higher than in the simulation but the discrepancies ranged from 3% up to almost 33%, at most (based on variant: CORRECT_GEOM_2). Firstly, it can be justified because in general, a realistic shape of paperboard can differ in the details from the numerical (idealized) model. Secondly, in the present simulation, the influence of the adhesive connecting all the layers of paper was not taken into account. This effect cannot be so significant, but the mentioned factors might have had an influence on the final scores.

## Figures and Tables

**Table 1:** Mechanical material data.

Model Number	Layer Number	Thickness (mm)	*E*_MD_ (GPa)	*E*_CD_ (GPa)	*n*_MD_ (-)	*n*_CD_ (-)	*G*_MD-CD_ (GPa)
Model_1	1-flat (*t*_1_)	0.142	5.70	2.24	0.118	0.3	2.06
	E-wave (*t*_E_)	0.164	5.73	1.56	0.082	0.3	1.73
	3-flat (*t*_2_)	0.126	6.46	1.97	0.091	0.3	2.06
	B-wave (*t*_B_)	0.164	5.73	1.56	0.082	0.3	1.73
	5-flat (*t*_3_)	0.146	5.65	2.47	0.131	0.3	2.16
Model_2	1-flat (*t*_1_)	0.185	6.69	2.50	0.112	0.3	2.36
	E-wave (*t*_E_)	0.227	5.49	1.87	0.102	0.3	1.85
	3-flat (*t*_2_)	0.177	5.20	1.80	0.104	0.3	1.77
	B-wave (*t*_B_)	0.199	6.64	1.95	0.088	0.3	2.08
	5-flat (*t*_3_)	0.186	5.52	1.86	0.101	0.3	1.85
Model_3	1-flat (*t*_1_)	0.142	5.7	2.24	0.118	0.3	2.06
	E-wave (*t*_E_)	0.199	6.64	1.95	0.088	0.3	2.08
	3-flat (*t*_2_)	0.126	6.46	1.97	0.091	0.3	2.06
	B-wave (*t*_B_)	0.139	5.72	2.13	0.112	0.3	2.02
	5-flat (*t*_3_)	0.146	5.65	2.47	0.131	0.3	2.16
Model_4	1-flat (*t*_1_)	0.142	5.70	2.24	0.118	0.3	2.06
	E-wave (*t*_E_)	0.177	5.20	1.80	0.104	0.3	1.77
	3-flat (*t*_2_)	0.139	5.72	2.13	0.112	0.3	2.02
	B-wave (*t*_B_)	0.177	5.20	1.80	0.104	0.3	1.77
	5-flat (*t*_3_)	0.146	5.65	2.47	0.131	0.3	2.16
Model_5	1-flat (*t*_1_)	0.185	6.69	2.50	0.112	0.3	2.36
	E-wave (*t*_E_)	0.199	6.64	1.95	0.088	0.3	2.08
	3-flat (*t*_2_)	0.177	5.20	1.80	0.104	0.3	1.77
	B-wave (*t*_B_)	0.199	6.64	1.95	0.088	0.3	2.08
	5-flat (*t*_3_)	0.186	5.52	1.86	0.101	0.3	1.85
Model_6	1-flat (*t*_1_)	0.142	5.70	2.24	0.118	0.3	2.06
	E-wave (*t*_E_)	0.177	5.20	1.80	0.104	0.3	1.77
	3-flat (*t*_2_)	0.164	5.73	1.56	0.082	0.3	1.73
	B-wave (*t*_B_)	0.177	5.20	1.80	0.104	0.3	1.77
	5-flat (*t*_3_)	0.186	5.52	1.86	0.101	0.3	1.85

**Table 2:** BS for all considered models.

Variant	EXP				FEM_1 NOMINAL_GEOM_1 (Nm)	FEM_1 CORRECT_GEOM_1 (Nm)	FEM_2 CORRECT_GEOM_2 (Nm)	FEM_1 NOMINAL Decrease (+)/ Increase (−) with Respect Mean Value (%)	FEM_1 CORRECT Decrease (+)/ Increase (−) with Respect Mean Value (%)	FEM_2 CORRECT Decrease (+)/ Increase (−) with Respect Mean Value (%)
	1 (Nm)	2 (Nm)	3 (Nm)	Mean Value (Nm)						
Model_1_B	8.50	8.20	8.25	8.32	5.80	6.98	7.62	30.29	16.11	8.41
Model_1_E	8.43	8.48	8.50	8.47	5.70	6.89	7.58	32.70	18.65	10.51
Model_2_B	11.10	11.00	10.80	10.97	7.47	9.04	9.88	31.91	17.59	9.94
Model_2_E	11.45	11.50	11.80	11.58	7.43	8.96	9.81	35.84	22.63	15.28
Model_3_B	8.67	6.96	6.13	7.25	5.76	7.01	7.61	20.55	3.31	−4.97
Model_3_E	9.65	9.40	9.45	9.50	5.72	6.89	7.53	39.79	27.47	20.74
Model_4_B	9.01	9.17	9.13	9.10	5.68	6.88	7.53	37.58	24.40	17.25
Model_4_E	10.95	11.10	11.25	11.10	5.63	6.80	7.45	49.28	38.74	32.88
Model_5_B	11.47	11.51	11.43	11.46	7.88	9.52	10.42	31.24	16.93	9.08
Model_5_E	12.70	13.25	12.95	12.97	7.83	9.46	10.37	39.63	27.06	20.05
Model_6_B	8.20	8.10	8.30	8.20	6.39	7.73	8.45	22.07	5.73	−3.05
Model_6_E	9.25	9.00	9.10	9.12	6.35	7.67	8.40	30.37	15.90	7.89

**Table 3:** Total displacement maps attained numerically for Model_1 (compressed B-wave).

Force *F* (N)	FEM_1 CORRECT_GEOM_1	FEM_2 CORRECT_GEOM_2
1	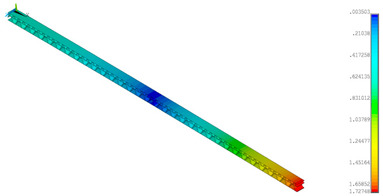	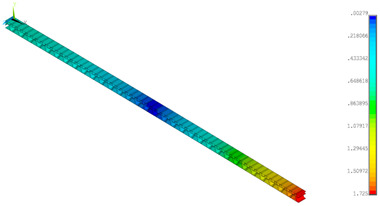
2	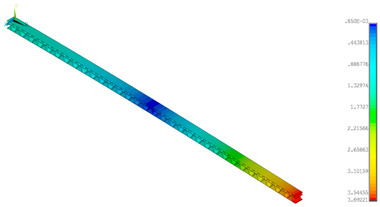	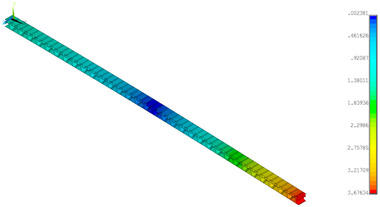
4	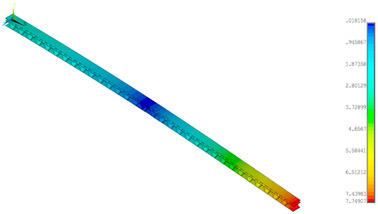	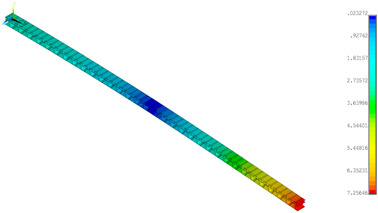
8	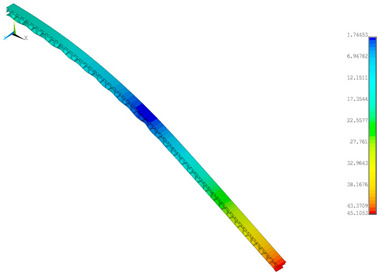	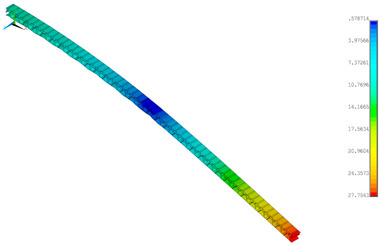

**Table 4:** Total displacement maps attained numerically for Model_1 (compressed E-wave).

Force *F* (N)	FEM_1 CORRECT_GEOM_1	FEM_2 CORRECT_GEOM_2
1	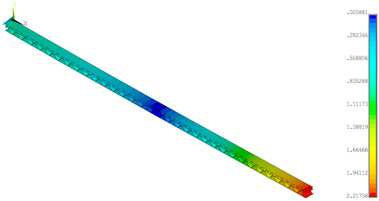	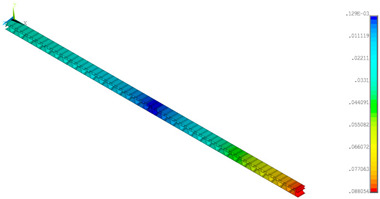
2	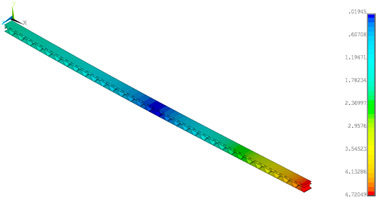	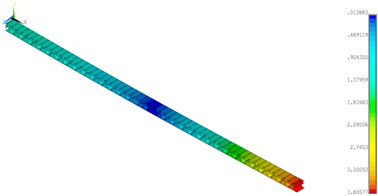
4	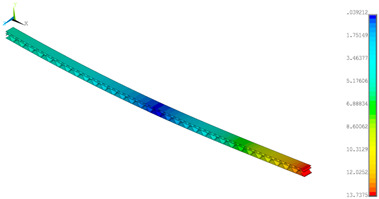	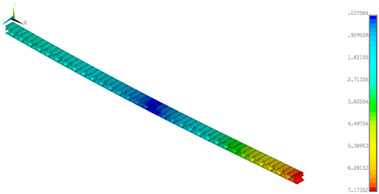
8	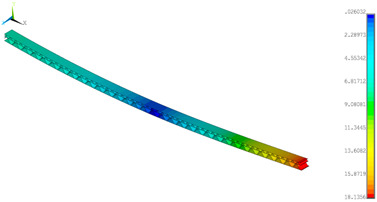	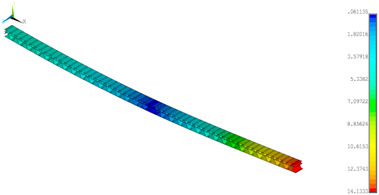

**Table 5:** Deformations of the corrugated cardboard sample under different force values during the experiment (compressed B-wave).

Point/ Force (N)	View	Magnified View of Bent Paperboard
B1 (2)	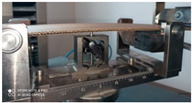	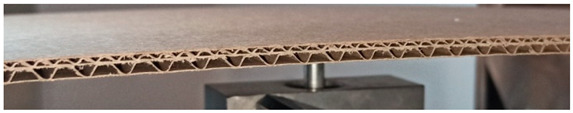
B2 (3)	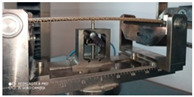	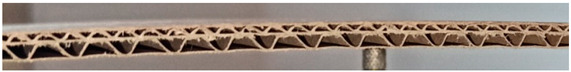
B3 (5)	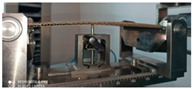	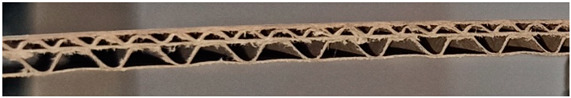
B4 (6)	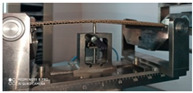	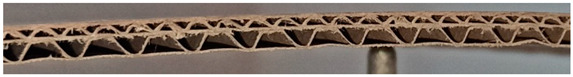
B5 (7)	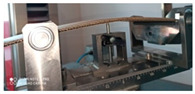	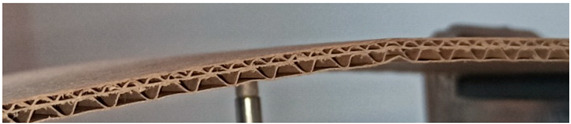

**Table 6:** Deformations of the corrugated cardboard sample under different force values during the experiment (compressed E-wave).

Point/ Force (N)	View	Magnified View of Paperboard
E1 (2)	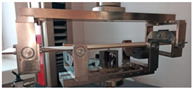	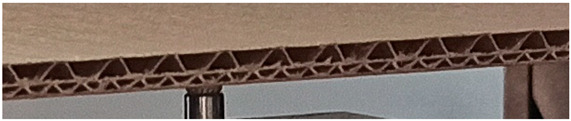
E2 (3)	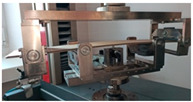	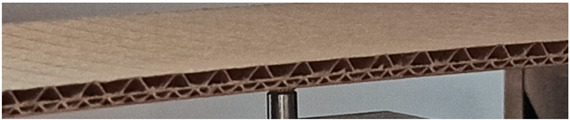
E3 (5)	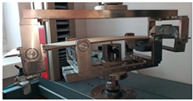	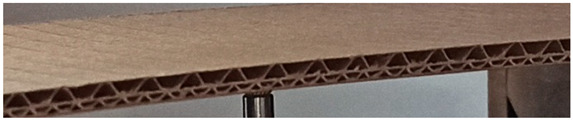
E4 (6)	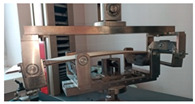	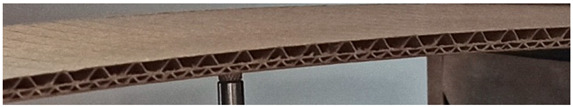
E5 (10)	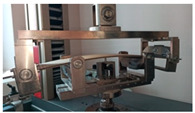	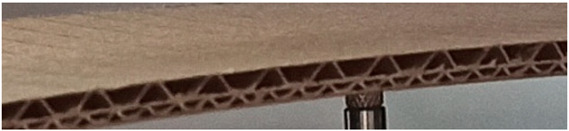
E6 (16.5)